# Genomics Analysis of Metabolic Pathways of Human Stem Cell-Derived Microglia-Like Cells and the Integrated Cortical Spheroids

**DOI:** 10.1155/2019/2382534

**Published:** 2019-11-18

**Authors:** Julie Bejoy, Xuegang Yuan, Liqing Song, Thien Hua, Richard Jeske, Sébastien Sart, Qing-Xiang Amy Sang, Yan Li

**Affiliations:** ^1^Department of Chemical and Biomedical Engineering, FAMU-FSU College of Engineering, Florida State University, Tallahassee, FL, USA; ^2^Department of Chemistry and Biochemistry, Florida State University, Tallahassee, Florida, USA; ^3^Hydrodynamics Laboratory (LadHyX)-Department of Mechanics, Ecole Polytechnique, CNRS-UMR7646, 91128 Palaiseau, France; ^4^Institute of Molecular Biophysics, Florida State University, Tallahassee, Florida, USA

## Abstract

Brain spheroids or organoids derived from human pluripotent stem cells (hiPSCs) are still not capable of completely recapitulating *in vivo* human brain tissue, and one of the limitations is lack of microglia. To add built-in immune function, coculture of the dorsal forebrain spheroids with isogenic microglia-like cells (D-MG) was performed in our study. The three-dimensional D-MG spheroids were analyzed for their transcriptome and compared with isogenic microglia-like cells (MG). Cortical spheroids containing microglia-like cells displayed different metabolic programming, which may affect the associated phenotype. The expression of genes related to glycolysis and hypoxia signaling was increased in cocultured D-MG spheroids, indicating the metabolic shift to aerobic glycolysis, which is in favor of M1 polarization of microglia-like cells. In addition, the metabolic pathways and the signaling pathways involved in cell proliferation, cell death, PIK3/AKT/mTOR signaling, eukaryotic initiation factor 2 pathway, and Wnt and Notch pathways were analyzed. The results demonstrate the activation of mTOR and p53 signaling, increased expression of Notch ligands, and the repression of NF-*κ*B and canonical Wnt pathways, as well as the lower expression of cell cycle genes in the cocultured D-MG spheroids. This analysis indicates that physiological 3-D microenvironment may reshape the immunity of *in vitro* cortical spheroids and better recapitulate *in vivo* brain tissue function for disease modeling and drug screening.

## 1. Introduction

Understanding the models established by human induced pluripotent stem cells (hiPSCs) requires genome-wide mapping to elucidate gene regulatory networks [1, 2]. Therefore, transcriptome analysis has been used to compare hiPSC-derived lineage-specific cells with somatic counterparts [3]. Recently, forebrain spheroids or organoids were derived from hiPSCs for disease modeling and as potential platforms for drug screening [4–7]. These spheroids need to contain critical components of the human brain, such as vascular cells and microglia, for proper function. Our previous study characterized microglia-like cells differentiated from hiPSCs and introduced isogenic microglia-like cells into forebrain spheroids [8]. The microglia-like cells were cocultured with isogenic dorsal cortical spheroids in order to build immune function within the spheroids. While extensive phenotypic characterizations were performed in our previous study, the fundamental metabolic pathways and signaling pathways in different culture systems were not analyzed yet. It is postulated that the microglia-like cells inside the spheroids retain more *in vivo*-like metabolic program and the associated phenotype due to the environmental change in intimate cell-cell and cell-matrix interactions compared to 2-D culture.

Metabolic programming was found to play important roles in homeostasis, tissue repair, immune function, epigenetic change, and cellular phenotype. For hiPSC reprogramming, the somatic cells with oxidative phosphorylation (OXPHOS) gain the glycolysis metabolism when they gain pluripotency [9, 10]. For human mesenchymal stem cell (MSC) aggregates, metabolic reconfiguration towards glycolysis supports reacquisition of the primitive stem cell phenotype [11]. Similarly, for microglia, limited data suggest that polarization to an M1 phenotype (proinflammatory) may be accompanied by a metabolic shift from oxidative phosphorylation to aerobic glycolysis for energy production [12, 13]. It has been suggested that default polarization of resident microglia uses OXPHOS (mitochondrial respiration, i.e., anti-inflammatory M2 phenotype) for the functions involved in tissue repair, while the metabolic program shifts towards glycolysis as well as nitric oxide and citrulline production for M1 polarization [12]. In addition, the metabolic environment of microglia can affect brain region-dependent gene regulation [13]. Therefore, modifications of immune response by the physiological 3-D microenvironment may reshape the innate immunity that gains the “memory” and “trained” inflammation response [12].

Based on the literature for metabolic programming of 3-D HepG2/C3A spheroids [14], this present study analyzed the genomics data for hiPSC-derived microglia-like cells (i.e., single cells in semiadherent culture) and the isogenic 3-D dorsal spheroids containing microglia-like cells. The focus is on changes in metabolic pathways and the signaling pathways that are involved in cell proliferation, cell death, inflammation, PIK3/AKT/mTOR signaling, and Wnt and Notch pathways in 3-D cortical spheroids derived from hiPSCs. This study provides global genomic details on the impact of 3-D culturing on the metabolic phenotype of cells inside the cortical spheroids. The analysis in this study can also help to establish better coculturing strategies for mimicking the *in vivo* structure and functions of the central nervous system *in vitro*.

## 2. Materials and Methods

### 2.1. Microglia-Like Cells Derived from iPSCs

**Figure 1:**
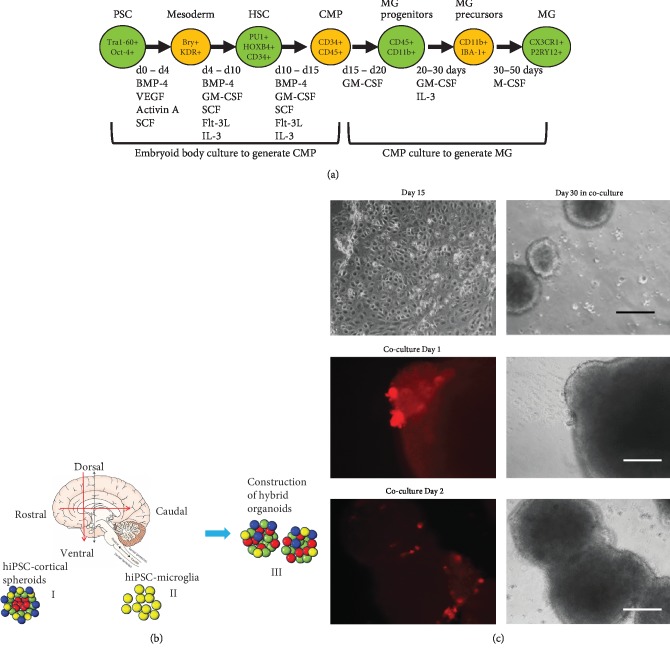
Illustration of the MG group and D-MG group. (a) Derivation of microglia-like cells (MG) from hiPSCs. (b) Incorporation of MG into the isogenic dorsal spheroids (DMG). (c) Morphology of the cells in microglial differentiation and the D-MG spheroids. Day 30 MGs were labeled with CellTracker Red. White scale bar: 100 *μ*m. Black scale bar: 200 *μ*m.

Our lab has derived microglia-like cells from human iPSK3 cells free of feeders using a modified protocol [15]. Mesoderm induction was performed using activin A, BMP4, SCF, and VEGF, followed by SCF, Flt3L, IL-3, and GM-CSF treatment. Nonadherent cells were replated to tissue culture-treated plates in the presence of IL-3 and M-CSF. The day 23 cells expressed 48-59% CD11b and 52-64% CD45. Day 28 cells expressed 70 ± 6% CD11b and 80 ± 5% IBA-1. Day 30-33 cells expressed 68 ± 12% P2RY12 and 51 ± 10% CX3CR1 (Figure 1(a)) [8].

### 2.2. Generation of Isogenic Dorsal Cortical Spheroids and Coculturing with Microglia-Like Cells

Neural differentiation was induced using dual SMAD inhibitors LDN193189 (LDN) and SB431542 (SB) on human iPSK3 cells in low attachment 96-well plates. The neural progenitor spheres were treated with patterning factors cyclopamine (a sonic hedgehog inhibitor) and fibroblast growth factor- (FGF-) 2 [7, 16]. The dorsal identity is defined by TBR1, PAX6, BRN2, and SATB2.

Dorsal spheroids (day 30) were cocultured with isogenic microglia-like cells at a 4 : 1 ratio (8 × 10^5^ neurons to 2 × 10^5^ microglia-like cells, i.e., 20%) in 50% DMEM/10% FBS and 50% neural differentiation medium composed of DMEM/F12 plus 2% B27 (Figures 1(b) and 1(c)). The CellTracker Red-labeled MGs were observed to move into the D-MG spheroids (Figure 1(c)). After three days of coculture (day 33), the dorsal spheroids containing microglia-like cells (D-MG group) and the microglia cells only (MG group) were harvested for RNA isolation and RNA-sequencing.

### 2.3. RNA-Sequencing

mRNA was isolated from the total RNA using the NEBNext Poly(A) mRNA Magnetic Isolation Module (New England Biolabs). cDNA libraries were generated from the isolated mRNA using an NEBNext Ultra RNA Library Prep Kit for Illumina (New England Biolabs), with a unique 6-nucleotide index primer (NEBNext Multiplex Oligos for Illumina), using Beckman Biomek 4000. The multiplexed sample was quantified with qPCR (Kapa Biosystems) specific for Illumina sequencing primers, and the average fragment size was determined with a Bioanalyzer High-Sensitivity DNA Chip (Agilent Technologies). The sequencing was performed on Illumina HiSeq 2500 at the Translational Science Laboratory of Florida State University. The pooled data were demultiplexed into individual sample data, and adapter primer sequences were removed [17].

### 2.4. RNA-Sequencing Data Analysis

The sequencing reads were analyzed using RNA-Seq Alignment version 1.1.1 (Illumina BaseSpace application). The reads were aligned with TopHat 2 [18] to the human genome (genome release GRCh38) using default parameters, and counts for each gene were generated. This workflow uses Cufflinks to generate FPKM (fragments per kilobase per million reads) normalized values [19]. DESeq2 was used to determine statistically significant differentially expressed genes (a false discovery rate (FDR) of <0.05 was used). 15,585 genes were considered to be expressed in this study by the DESeq2 software [20]. The top 500 genes that were upregulated and downregulated (1000 total genes) in the microglial culture versus the D-MG group were assessed for GO, KEGG pathway, and phenotype pathway analysis using WebGestalt [21, 22]. Significant enrichment was determined in WebGestalt using the hypergeometric test and the Benjamini-Hochberg FDR method [23] for multiple testing adjustment.

### 2.5. Reverse Transcription Polymerase Chain Reaction (RT-PCR) Analysis

Total RNA was isolated from different cell samples using the RNeasy Mini Kit (Qiagen, Valencia, CA) according to the manufacturer's protocol followed by the treatment with a DNA-Free RNA Kit (Zymo, Irvine, CA) [24]. Reverse transcription was carried out using 2 g of total RNA, anchored oligo-dT primers (Operon, Huntsville, AL), and Superscript III (Invitrogen, Carlsbad, CA) (according to the protocol of the manufacturer). Primers specific for target genes (Supplementary [Supplementary-material supplementary-material-1]) were designed using the software Oligo Explorer 1.2 (GeneLink, Hawthorne, NY). The gene *β*-actin was used as an endogenous control for normalization of expression levels. Real-time RT-PCR reactions were performed on an ABI7500 instrument (Applied Biosystems, Foster City, CA), using SYBR1 Green PCR Master Mix (Applied Biosystems). The amplification reactions were performed as follows: 2 min at 50°C, 10 min at 95°C, and 40 cycles of 95°C for 15 sec and 55°C for 30 sec and 68°C for 30 sec. Fold variation in gene expression was quantified by means of the comparative Ct method: 2^−(Δ*C*_*t* treatment_ − Δ*C*_*t* control_)^, which is based on the comparison of expression of the target gene (normalized to the endogenous control *β*-actin) between the compared samples.

## 3. Results and Discussions

Previously, our study derived and characterized microglia-like cells from the healthy iPSK3 cells and Ep-iPSC cells [8]. The cells were cocultured and integrated with isogenic dorsal cortical spheroids (Supplementary [Supplementary-material supplementary-material-1]). The whole-cell transcriptome analysis was performed for the microglial phenotype, neural inflammation, and the genes involved in Alzheimer's disease. Our current study focuses on different attributes of the 3-D dorsal cortical spheroids containing isogenic microglia-like cells (D-MG group) vs. microglia-like cells (MG group), including metabolic pathways and the signaling pathways that are involved in cell proliferation, cell death, inflammation, PIK3/AKT/mTOR signaling, EIF2 pathway, and Wnt and Notch pathways, which were not reported previously.

### 3.1. Central Metabolic Pathway

The 3-D spheroid culture usually is associated with slow growth rates, cytoskeleton reorganization, enhanced tight junctions and polarity, and the relocated membrane transporters [25–27]. In aerobic glycolysis, glucose is consumed through the glycolytic pathway to produce lactic acid, nucleotides, amino acids, and other metabolites, while glutamine is converted through glutaminolysis to citrate for cholesterol and lipid production. In oxidative phosphorylation, the generated pyruvate is oxidized to CO_2_ and water through the tricarboxylic (TCA) cycle, which produces ATP and coverts NADH to NADPH [28–30].

**Figure 2:**
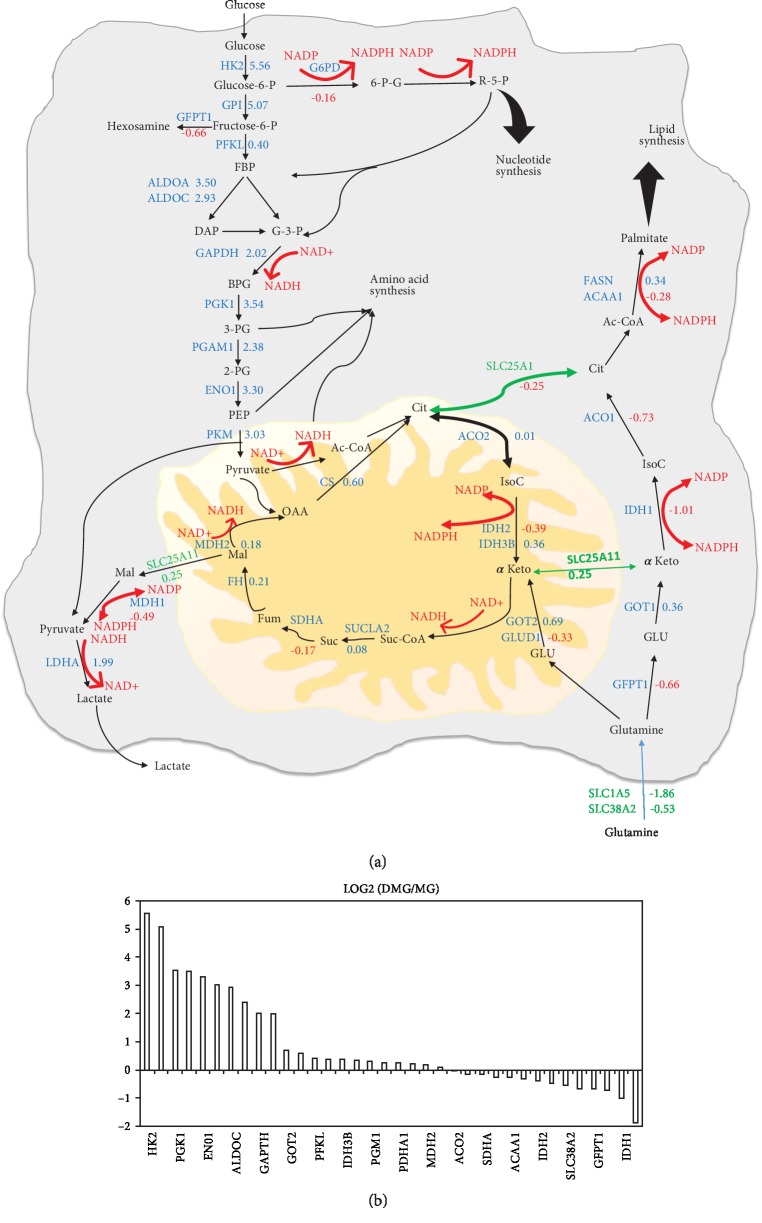
Ratios of gene abundance of central metabolic pathways in the D-MG group in comparison to the MG group. The FPKM (fragments per kilobase per million reads) normalized values for these genes are listed for both samples. The numbers are the log2 values of ratios of D-MG to MG. Negative values indicate that the genes are present in higher amounts in the MG group, while positive values indicate that the genes are present in higher amounts in the D-MG group. (a) Central metabolic pathways. (b) Log2 values of glycolysis genes of D-MG/MG ratios.

The central metabolic pathways are shown in Figure 2. The values were calculated using log2 (DMG/MG). Relative enzyme expression levels indicate an increase in glycolytic and pentose phosphate pathways in the D-MG group as well as the increased amino acid synthesis (Figure 2 and Supplementary [Supplementary-material supplementary-material-1]) [31]. The increased glycolysis is permissive to trigger microbiocidal activity of microglia and allows the cells to survive in the 3-D spheroids. However, enzymes in the glutaminolytic pathway, hexosamine, nucleotide, lipid synthesis, and TCA cycle have small differences among the two groups. Genes related to ATP synthesis and mitochondrial complexes I, III, and IV are shown in Supplementary [Supplementary-material supplementary-material-1] and Supplementary [Supplementary-material supplementary-material-1]. The differences between the two groups are small (within ±0.5 except NDUFA13 at -0.68). But the values are all negative for genes related to ATP synthesis and mitochondrial complexes I and III, indicating lower expression in the D-MG group. Together, these results indicate the different energetic/metabolic phenotypes between the D-MG and the MG groups.

Metabolic shift towards glycolysis usually results in the elevated dependence on glutaminolysis or fatty acid synthesis in cell metabolism [32]. However, in this study, the cells did not show a significant increase in glutaminolysis in the spheroids (Figure 2 and Supplementary [Supplementary-material supplementary-material-1]). Cytoplasmic isocitrate dehydrogenase 1 (IDH-1) is involved in the reaction of isocitrate production from *α*-ketoglutarate in the cytosol. IDH-1 expression is decreased in the D-MG spheroids, which may be associated with NF-*κ*B activation in a hypoxia-inducible factor- (HIF-1*α*-) dependent manner [33].

Similarly, 3-D culture of iPSC-derived endothelial cells also showed the glycolysis-dominated metabolism compared to ones in 2-D culture [34]. Moreover, human MSCs treated with interferon gamma exert an immunosuppressive phenotype by secreting PGE2/IDO and reconfigure the metabolic phenotype to aerobic glycolysis [35]. It was also suggested that activation of the AKT/mTOR signaling pathway is required for metabolic shift under this immune polarization.

The 3-D cultures may not always enhance the glycolysis, which is dependent on a specific cell type. For example, it was reported that 3-D cultures of hiPSC-derived cardiomyocytes displayed downregulation of genes involved in glycolysis and lipid biosynthesis and upregulation of genes involved in oxidative phosphorylation. Accordingly, the 3-D cultures showed lower fluxes for fatty acid synthesis and the increased TCA cycle activity, which improved both cell purity and metabolic maturation [36].

### 3.2. Hypoxia (Glycolysis and Oxidative Phosphorylation)

#### 3.2.1. HIF-1*α* Pathway

**Figure 3:**
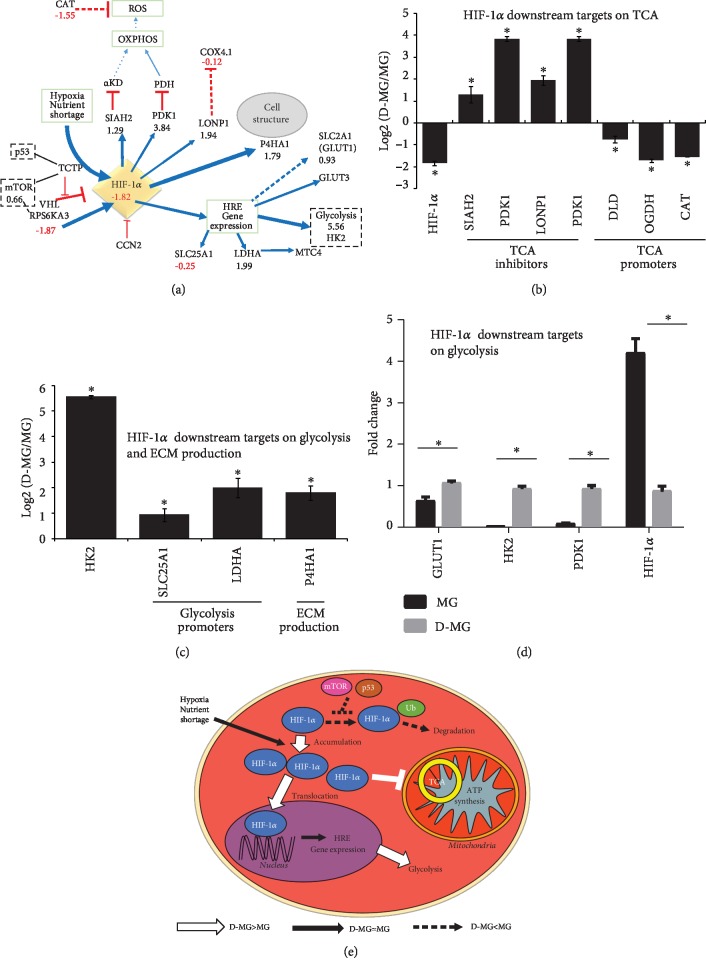
HIF-1*α*-related pathway. (a) RNA-sequencing results for glycolytic genes and HIF-1*α*; relative transcript expression between D-MG and MG for HIF-1*α* and its downstream targets (b) on tricarboxylic cycle (TCA) and (c) on glycolysis and extracellular matrix (ECM) production. ∗ indicates *p* < 0.05 (*n* = 3). (d) Validation of glycolytic genes using RT-PCR. ∗ indicates *p* < 0.05 (*n* = 3). (e) Schematic diagram showing the major changes in D-MG versus MG for HIF-1*α* signaling: D-MG shows enhanced HIF-1*α* activities that reduce TCA and ATP production, increasing glycolysis mediated by HIF-1*α*.

In 3-D spheroid culture, the inside of the spheroids is thought to be more hypoxic than the surface due to mass transfer limitation of oxygen [37], while this has been challenged by other studies as nonhypoxia-stabilized HIF expression [25]. Hypoxia is an important factor in regulating stem cell metabolism and phenotype [38]. When oxygen concentrations decrease, the oxygen-dependent prolyl hydroxylase domain proteins are inactivated and the HIF-1*α* protein is accumulated, which promotes HIF-1*α* translocation to the nucleus and its binding to hypoxia response elements, such as glucose transporters and glycolytic enzymes [39, 40]. Our results do not show the higher HIF-1*α* gene expression in the D-MG group but demonstrate the increased expression of HIF-1*α* pathway downstream genes, including SIAH2 (1.29), PDK1 (3.84), LDHA (1.99), LONP1 (1.94), and P4HA1 (1.79) (Figures 3(a)–3(c)). These results may indicate the nonhypoxia-stabilized HIF expression in the D-MG group. The downregulated HIF-1*α* gene expression in the D-MG group was also validated using RT-PCR, along with the upregulated glycolytic gene expression in the D-MG group (Figures 3(d) and 3(e)).

HIF-1*α* induces pyruvate dehydrogenase kinase 1 (PDK1) expression, which inhibits mitochondrial pyruvate dehydrogenase (PDH) [38, 41]. This reduces pyruvate flux into the TCA cycle and lowers the mitochondrial oxygen requirements. The lactate production and secretion would be increased, as observed by Sart et al. [9]. HIF-1*α* also induces E3-ubiquitin ligase SIAH2 synthesis, which mediates the proteasomal degradation of the OGDH subunit of *α*-ketoglutarate dehydrogenase (*α*-KD) and forms a part of the feedback control of HIF-1*α* signaling. A modest reduction of the *α*-KD enzyme complex was observed in the D-MG group (DLD, -0.75, OGDH, -1.71) (Table 1), which may slow down TCA cycle activity. Interestingly, the citrate transportation into the cytoplasm by citrate transporter protein (SLC25A1, -0.25) was relatively comparable between these two groups. The extracellular matrix remodeling via collagen hydroxylases (P4HA1, 1.79) was upregulated in the D-MG group. The amounts of anabolic rate-limiting enzymes were increased or remained similar, while catabolic enzymes were unchanged between the two groups (Table 2). The rate-limiting glycolytic pathway steps (HK2, PKM, and PFKL) are the mostly increased enzymes of the pathway. These findings are consistent with the metabolic shift to aerobic glycolysis. In addition, high PFK level may also indicate high levels of AMP, since the spheroids usually have high ATP amounts [14].

HIF-1*α* signaling can induce the expression of the mitochondrial protease LONP1 (1.94) (Figure 3). LONP1 degrades cytochrome C oxidase 4 subunit 1 (COX4-1) through electron transport chain complex IV, allowing the replacement of COX4-1 by COX4-2 [42], which is more efficient in enzymatic reaction. LONP1 is an essential central regulator of mitochondrial activity and is overexpressed during oncogenesis [43]. Although LONP1 was increased (1.94) based on our results, there was little change in the level of COX4-1 (-0.12). Reduced mitochondrial respiration normally results in fewer reactive oxygen species (ROS), correlated with the reduced level of catalase (CAT, -1.55). The reduced oxidative stress results in the diminished hydrogen peroxide damage and less oxidized proteins [14].

#### 3.2.2. Glutamine Metabolism and Hexosamine Pathway

While the D-MG group mainly uses glycolysis as its major energetic metabolism, our results did not show an increased reliance on glutamine metabolism (Figure 2 and Supplementary [Supplementary-material supplementary-material-1]). Intracellular glutamine levels are regulated by plasma membrane transporters SLC38A2 and SLC1A5 [14]. Endoplasmic reticulum stress would induce the degradation of transporters and ultimately autophagy and cell death [14]. In D-MG spheroids of this study, both SLC38A2 (-0.53) and SLC1A5 (-1.86) were decreased, which may suggest enhanced autophagy in D-MG spheroids.

*(1) Conversion of Glutamine into Glutamate*. The glutamate demand of the cells is indicated by the expression of GFPT1 (-0.66). GFPT1 is the first and rate-limiting step of the hexosamine pathway and catalyzes the conversion of fructose 6-phosphate and glutamine to glucosamine 6-phosphate and glutamate. Consistently in this study, several enzymes in polysaccharide, proteoglycan, and glycosylation synthetic pathways were downregulated in the D-MG spheroids, including UDP-glucose pyrophosphorylase UGP2 (-0.51), UDP-glucose 6-dehydrogenase UGDH (-1.18), and UDP-glucose 4-epimerase GALE (-1.31).

*(2) α-Ketoglutarate*. Glutamate can be converted to *α*-ketoglutarate by the mitochondrial GLUD1 (-0.33) or by cytoplasmic alanine or aspartate aminotransferases [44]. The cytoplasmic enzymes were slightly upregulated (GOT1, 0.36, GOT2, 0.69) in the D-MG group. There are three possible routes by which *α*-ketoglutarate can be converted to citrate (Figure 2) [1]. It can be converted through the TCA cycle [2]. It could be converted from isocitrate to citrate (by IDH2, -0.39, and ACO2, 0.01), which affects the normal TCA cycle flux by reductive glutamate metabolism [32]. In this study, IDH3B was slightly increased (0.36) and it can only catalyze the “forward” reaction [3]. Cytoplasmic *α*-ketoglutarate, produced through GFPT1 (-0.66) and GOT1 (0.36), can be converted by IDH1 (-1.01) and ACO1 (-0.73), which are both downregulated in the D-MG group compared to the MG group.

*(3) NADH*. NADH/NAD+ and NADPH/NADP+ conversions are critical in the central metabolic pathways (Figure 2 and Supplementary [Supplementary-material supplementary-material-1]). The reduction in the mitochondrial MDH1 (-0.49) and the downregulation of NAD(P) transhydrogenase (NNT, -1.24) suggest that the conversions in mitochondria may have some significance. MDH1 was decreased (-0.49) but lacked a malate source (e.g., the SLC25A11 transporter was essentially unchanged (0.25)). The conversion of cytoplasmic pyruvate to lactate would consume the produced NADH. The richest source of NADPH is the pentose phosphate pathway, where G6PD (-0.16) was essentially unchanged between the two groups. These results indicate the reliance on mitochondrial NAD+/NADH in the D-MG group.

*(3) Citrate*. Citrate can be used for fatty acid synthesis. ATP-citrate synthase uses citrate to generate cytosolic acetyl-CoA. Acetyl-CoA is used for (1) histone acetylation by acetyl-CoA acyltransferase (ACAA1, -0.28), (2) fatty acid synthesis (FASN, 0.34), and (3) synthesis of cholesterol, steroid hormones, and other biomolecules. Glutamine is as important as glucose in metabolism, and it can block glutamate-dependent cellular pathways at either IDH1 or ACL step [14].

### 3.3. Signaling Pathways Involved in Metabolic Reprogramming

The analysis so far shows the metabolic shift to aerobic glycolysis in the 3-D spheroids, which may be driven by diffusion gradients [30, 37]. In order to investigate how metabolic reprogramming is orchestrated, the status of critical pathways for cellular function, including PI3K/AKT/mTOR, Myc, p53, NF-*κ*B, EIF2, Wnt, and Notch, was analyzed.

#### 3.3.1. PIK3/AKT/mTOR Signaling

**Figure 4:**
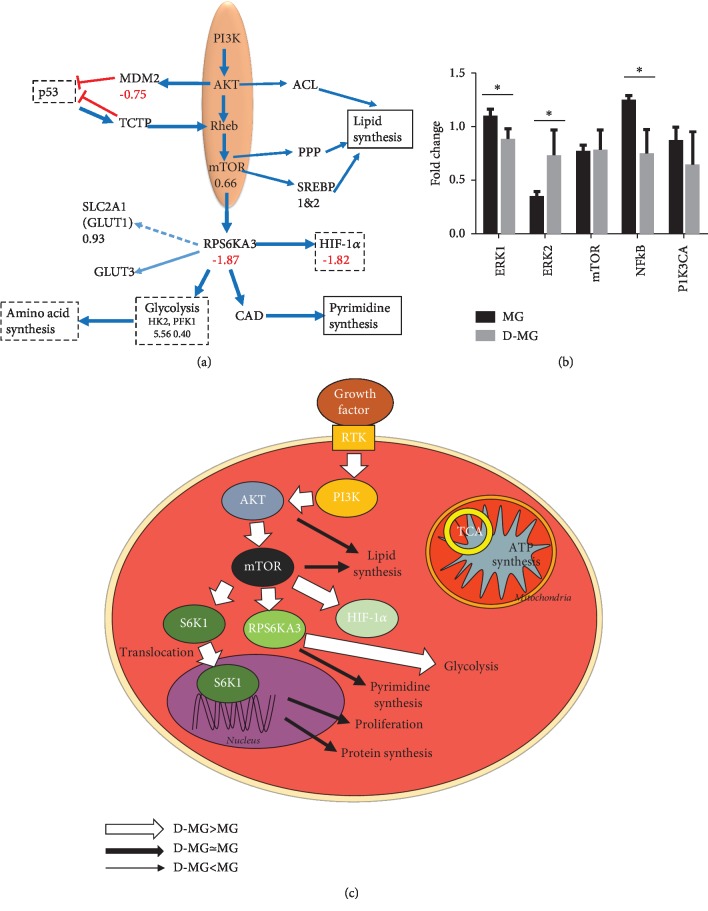
mTOR signaling in the D-MG group. (a) RNA-sequencing results. (b) Validation of ERK genes using RT-PCR. ∗ indicates *p* < 0.05 (*n* = 3). (c) Schematic diagram showing the major changes in D-MG versus MG condition for mTOR signaling. D-MG shows enhanced mTOR activities that reduce tricarboxylic cycle (TCA), and D-MG shows increased glycolysis mediated by mTOR.

PI3K (a class I phosphoinositide 3-kinase) is one of the key signaling enzymes that are activated in 3-D spheroid cultures [45]. And the PIK3 pathway is directly related to cellular metabolism and growth. When cells consume the nutrients and growth factors, they generate amino acids, glucose, oxygen, and energy (i.e., ATP) and use these products to activate GTPase, such as Rheb, through PI3K. One key regulator of metabolism and growth-activated downstream of PI3K signaling is the mechanistic target of rapamycin (mTOR) [46]. Our results showed that the expression of mTOR (0.66) was slightly elevated in the D-MG spheroids and the downstream effectors were inhibited (e.g., ribosomal protein s6 kinase RPS6KA3, -1.87) (Figure 4(a)). mTOR stimulates pyrimidine synthesis via the RPS6KA-mediated phosphorylation of CAD (0.46), whereas AKT can phosphorylate ACL, enhancing its lipogenic activities and mTOR signaling. The expression of ACLY (-0.13) indicates that the activity of the AKT pathway was similar in the two groups. mTOR regulates several anabolic and catabolic pathways through fatty acid and cholesterol biosynthetic genes via the SREBP family (SREBP1 and SREBP2) [47].

mTOR signaling was reported to increase the translation of HIF-1*α*, glucose transporters, and glycolytic enzymes and promote metabolic reprogramming. The activation of the mTOR protein complex mTORC1 leads to the induction of genes encoding the enzymes of glycolysis, the pentose phosphate pathway, and lipid and sterol biosynthesis [35, 48]. mTOR was also reported to coordinate the activation of cell growth machinery together with amino acids in the presence of growth signals [49]. However, in the RT-PCR validation, mTOR gene expression was statistically insignificant, possibly due to assay variations (Figures 4(b) and 4(c)). In addition, ERK1 was downregulated while ERK2 was upregulated in the D-MG group.

#### 3.3.2. Myc Signaling

**Figure 5:**
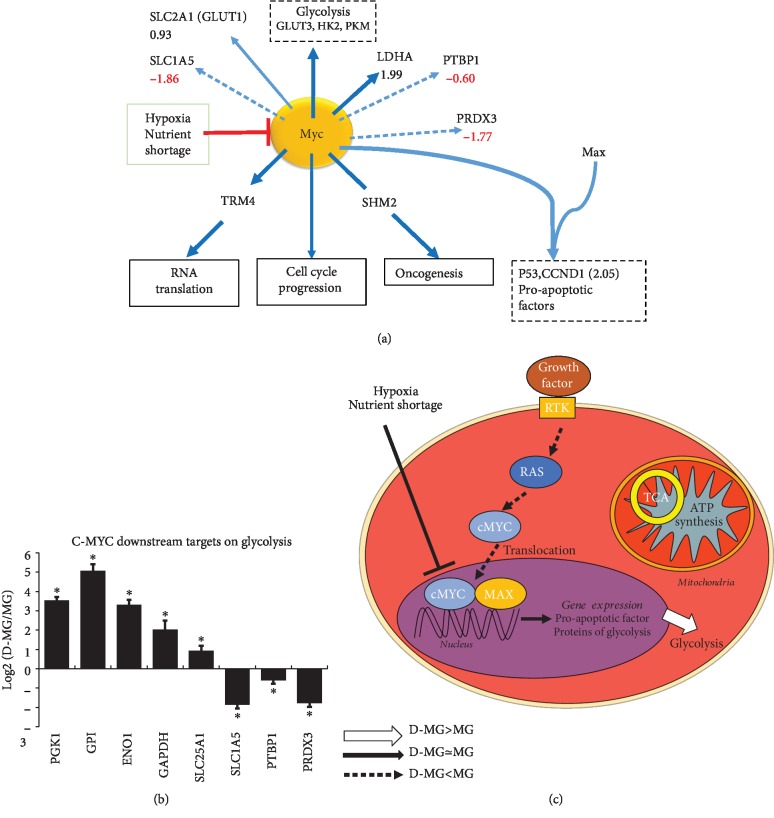
c-Myc signaling in the D-MG group. (a) RNA-sequencing results related to Myc. (b) Relative transcript expression between D-MG and MG conditions for c-Myc and its downstream targets; ∗ indicates *p* < 0.05 (*N* = 3). (c) Schematic diagram showing the major changes in D-MG versus MG for c-Myc signaling: D-MG shows enhanced c-Myc that mediates increased glycolysis.

Myc is a transcription factor that dimerizes with MAX to bind to DNA and regulate gene expression involved in metabolism (glycolysis and glutaminolysis) and biosynthesis (nucleotide and lipid synthesis) [50]. Myc supports specific mRNA splice variants, such as glycolysis-affecting PKM2 over PKM1. Myc targets glucose membrane transporters such as GLUT1 (or SLC2A1) and glutamine transporter SLC1A5, which are important in cell proliferation [50]. Myc transactivates the gene expression of PFK, ENO, and LDHA (1.99) and indirectly increases GAPDH and PGK1, based on our genomics analysis (PGK1, 3.54; GPI, 5.07; ENO1, 3.30; and GAPDH, 2.02), indicating the relation between Myc and glycolysis (Figure 5). The gene expression of Myc-related proteins was downregulated in the D-MG spheroids including PTBP1 (-0.60), SLC1A5 (-1.86), and PRDX3 (-1.77) (Figure 5). The low Myc activity inside the D-MG spheroids might indicate the slow proliferation of cells.

#### 3.3.3. p53 Signaling

**Figure 6:**
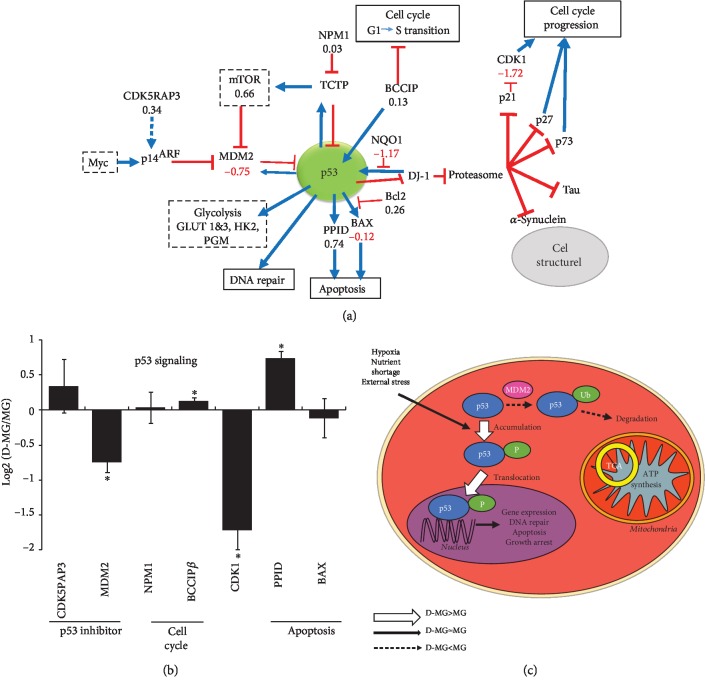
p53 signaling in the D-MG group. (a) RNA-sequencing results related to p53. (b) Relative transcript expression between D-MG and MG for p53 and its downstream targets; ∗ indicates *p* < 0.05 (*N* = 3). (c) Schematic diagram showing the major changes in D-MG versus MG for p53 signaling: D-MG shows the increased p53 activities, which results in the decreased expression of proteins related to cell proliferation.

In addition, the expression of the transcriptional suppressor CDK5RAP3 is comparable between the D-MG group and the MG group (0.34). CDK5RAP3 is a novel activator of PAK4 and processes important prometastatic function [51]. It has been reported that CDK5RAP3 knockdown upregulated the tumor suppressor p14ARF at protein and mRNA levels, and ectopic expression of CDK5RAP3 repressed the transcription of p14ARF [51]. Therefore, the CDK5RAP3 expression may allow for the synthesis of p14ARF and its binding to MDM2. The MDM2 (-0.75) expression was decreased in the D-MG spheroids (Figure 6). The MDM2 expression can release p53 from inhibition and thereby lead to the stabilization, accumulation, and activation of p53. Also, the negative regulators of p53 are subjected to tight feedback regulation. For example, BCCIP*β* overexpression delays the G1 to S progression and results in an elevated p21 expression, which would inhibit CDK1 induction of cell cycle progression. Though the D-MG spheroids did not show a significant increase in BCCIP*β* expression (0.13) compared to the MG group (Figure 6), the expression of CDK1 was decreased (-1.72) in the D-MG group, which indicated a potential cell cycle arrest.

#### 3.3.4. Wnt Pathway

**Figure 7:**
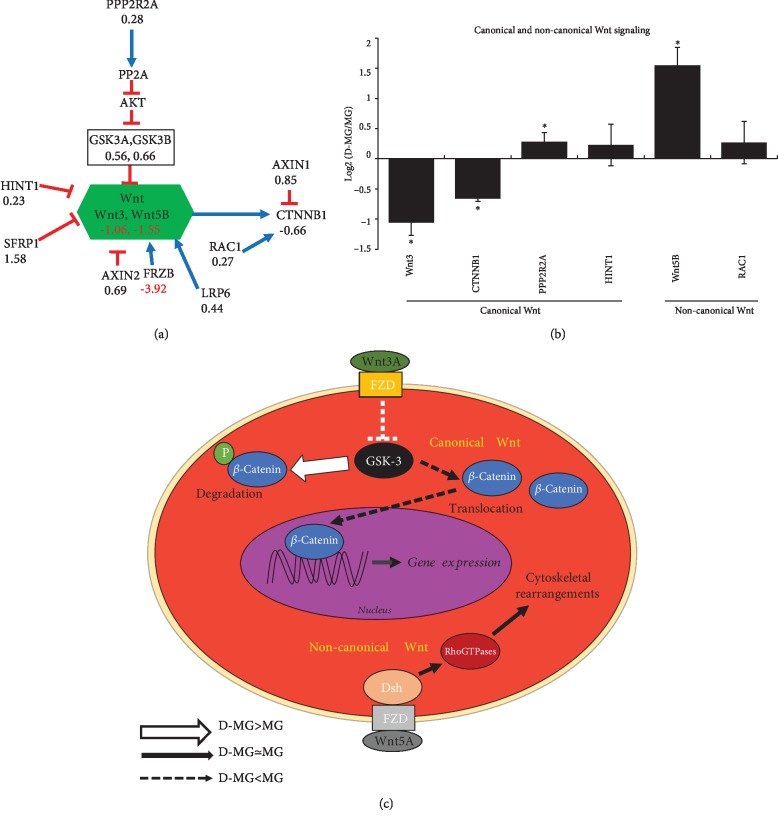
Wnt signaling in the D-MG group. (a) RNA-sequencing results related to Wnt. (b) Relative transcript expression between D-MG and MG for Wnt and its downstream targets. ∗ indicates *p* < 0.05 (*N* = 3). (c) Schematic diagram showing the major changes in D-MG versus MG conditions for Wnt signaling; i.e., D-MG shows the decreased canonical Wnt signaling while noncanonical Wnt is increased, which results in the increased cytoskeletal rearrangement.

In the canonical Wnt pathway, Wnt interacts with the Frizzled receptor that results in the inhibition of glycogen synthase kinase-3*β* (GSK-3*β*) [52]. Due to the inhibition, GSK-3*β* would not be able to phosphorylate *β*-catenin, leading to the nuclear entry of *β*-catenin. In the nucleus, *β*-catenin interacts with members of TCF transcriptional factors (i.e., T cell factor/lymphoid enhancer factor) and modulates target gene expression [53]. In our D-MG spheroids, the expression of *β*-catenin was slightly reduced compared to that in the MG group (CTNNB1, -0.66) (Figure 7). Wnt 3 (-1.06) and Wnt 5B (-1.55) were also decreased. The histidine triad nucleotide-binding protein 1 (HINT1, 0.23), which keeps the Wnt/*β*-catenin pathway inactive [54], and protein phosphorylase 2A (PPP2RA1, 0.28) were comparable in the two groups. Our previous studies evaluated the influence of the canonical Wnt pathway on neural patterning of hiPSCs [55, 56]. The reduced canonical Wnt/*β*-catenin signaling at a late stage of neural differentiation of hiPSCs can enrich cells with rostral forebrain identity [57]. In the noncanonical pathway, Wnt binds to the Frizzled receptor which activates Dishevelled and forms a complex with RAC1, mediating profilin binding to actin [58]. In the current study, the noncanonical Wnt pathway activity was comparable for the D-MG spheroids and MG group (RAC1, 0.27).

#### 3.3.5. NF-*κ*B Pathway

NF-*κ*B was found to be involved in cellular responses to various stimuli such as stress, cytokines, free radicals, and viral antigens [59, 60]. The two classes of NF-*κ*B proteins (p50/p52 and ReIA/ReIB) form heterodimers to function as transcriptional activators. In an inactive state, NF-*κ*B dimers are sequestered in the cytoplasm by IkB inhibitors. They mask the nuclear localization signals of NF-*κ*B proteins and keep NF-*κ*B signaling in the inactive state. When active, NF-*κ*B proteins enter the nucleus and turn on the IkB*α* repressor. It has been reported that hypoxia upregulated PRMT1 which asymmetrically methylated ReIA, inhibiting the binding of ReIA to DNA and further repressing NF-*κ*B [61]. NF-*κ*B signaling was found to be dysregulated in TGF*β*R3 epicardial cells, which also showed the impaired cell invasion and revealed the role of NF-*κ*B signaling in TGF*β*R3 activation [62]. In our D-MG spheroids, the lower expression of TGF*β*R3 (-2.61) and TGFB1 (-0.60) indicates the less activated NF-*κ*B pathway (Supplementary [Supplementary-material supplementary-material-1] and Figure 4(b)). TGFBI expression is essentially similar (0.03), and PRMT1 expression (0.63) is slightly upregulated.

Microglial M1 immune response usually involves NF-*κ*B signaling [59]. Knocking down of LRP1 in primary microglia led to the activation of both c-Jun N-terminal kinase and NF-*κ*B pathways. The sensitivity to lipopolysaccharide (LPS) stimulation in the production of proinflammatory cytokines was also enhanced. The NF-*κ*B inhibitor was shown not only to suppress the production of cytokines induced by the knockdown of LRP1 but also to restore the downregulated expression of LRP1 due to LPS stimulation.

#### 3.3.6. Notch Pathway

**Figure 8:**
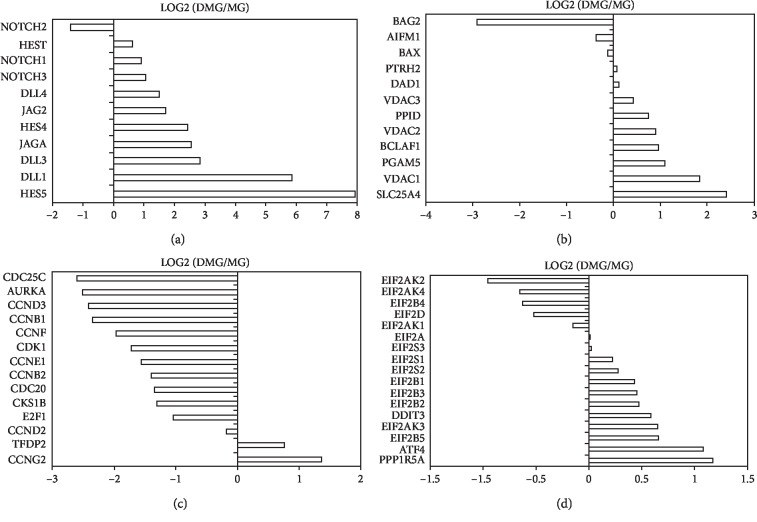
Log2 expression levels of the D-MG/MG ratios for genes about cell-cell interactions, cell death, and cell cycle. (a) Notch signaling. (b) Cell death. (c) Cell cycle and cell proliferation. (d) EIF2 signaling as a part of integrated stress response (ISR).

Most Notch receptors were upregulated in the D-MG group, including DLL1 (5.85), DLL3 (2.84), DLL4 (1.49), JAG1 (2.55), JAG2 (1.70), HES1 (0.62), HES4 (2.44), and HES5 (7.93) (Figure 8(a)), indicating the enhanced cell-cell communications in 3-D culture. Our previous study also showed that Notch-1 expression was upregulated in the hybrid stem cell spheroids [63]. In addition, Notch signaling regulates the balance between the progenitor pool and the neuron pool, preventing premature neurogenesis [64]. Global deletion of Notch signaling leads to the accelerated differentiation into neurons. The activation of Notch by DLL1 causes neural stem cells to irreversibly commit to a glial fate and prevents the stem cells from adopting a neuronal fate [65]. When a Notch receptor interacts with its ligands, Delta and Serrate, it activates the membrane-tethered transcription factor and leads to the release of intracellular domain (NICD) and its translocation to the nucleus. Then, NICD interacts with CSL family regulators and transcript Hes1 and Hes5 expression. Notch signaling also promotes Wnt expression in Drosophila development [66]. Conversely, Wnt protein can promote the expression of Notch ligands DLLs, forming a positive feedback loop to maintain Notch signaling and Wnt protein expression.

#### 3.3.7. Cell Death and Cell Cycle

It is important to evaluate cell death pathways (necrosis or apoptosis) in the 3-D spheroids. The major changes associated with cell death are free radical damage, swelling, rupture, and cytolysis. Under stress, the cells sense DNA damage by the serine/threonine kinase ATM and p53 [67]. p53 accumulates in the mitochondrial matrix and triggers mitochondrial permeability transition (MPT) pore (PTP) opening. Then, p53 physically interacts with anti- and proapoptotic Bcl-2 and BAX family members to inhibit or activate their respective functions, leading to mitochondrial outer membrane permeabilization (MOMP) and apoptosis [68]. From our results, PPID (0.74), the essential component of the MPT pore located in the mitochondrial matrix, is slightly increased in the D-MG group (Figure 8(b)).

The exact molecular composition of the MOMP complex is assumed to contain hexokinase, voltage-dependent anion channel (VDAC, on the outer membrane), the adenine nucleotide translocase (ANT, in the inner membrane), and cyclophilin D (a peptidyl-prolyl isomerase in the matrix). When PTP opens, it leads to matrix swelling and depolarization of the membrane potential, causing subsequent rupture of the outer membrane and consequently apoptosis [68]. Based on our results, VDACs were increased (VDAC1, 1.84; VDAC3, 0.90) in the D-MG group (Figure 8(b)). MPT-driven necrosis works not only by the dissipation of mitochondrial transmembrane potential but also by the latent chromatinolytic activity of AIFM1 (apoptosis-inducing factor mitochondrion-associated 1) [69]. The slightly decreased AIFM1 (-0.38) in the D-MG spheroids indicates that necrosis is favored over apoptosis.

The mitochondrial HINT2 promotes angiogenesis via p53 and BAX. The activated NF-*κ*B pathway also leads to the increased expression of antiapoptotic proteins, including Bcl-XL-binding protein v68 (PGAM5, 1.09) and Bcl-2-associated transcription factor 1 (BCLAF1, 0.96) in the D-MG spheroids (Figure 8(b)). The defender against apoptotic cell death (DAD1, 0.11) and Bcl-2 inhibitor of transcription 1 (PTRH2, 0.07) were unchanged. These antiapoptotic proteins can bind to and inactivate proapoptotic proteins. The proapoptotic protein BAX, Bcl-2-like protein 4, was unchanged (-0.02), but Bcl-2-associated athanogene 2 (BAG2, -2.90) was decreased in the D-MG spheroids. Consistent with higher expression of death genes in the D-MG group, the cell cycle-related genes were downregulated in the D-MG spheroids but highly expressed in the MG group (Figure 8(c)).

The sirtuin signaling proteins SIRT1 (0.48) and SIRT6 (1.01) were upregulated in the D-MG spheroids (Table 3), which regulate DNA damage and oxidative stress. The autophagy-related genes were essentially similar between the two groups. Poly (ADP-ribose) polymerase (PARP) is a family of proteins involved in a number of cellular processes such as DNA repair, genomic stability, and programmed cell death [70]. PARP6 (1.32), PARP3 (1.12), and PARP2 (0.97) were upregulated while PARP4 (-1.34), PARP14 (-2.44), PARP12 (-2.54), and PARP9 (-2.76) were downregulated in the D-MG group (Table 4). PARP1 (-0.01) was essentially unchanged between the two groups.

#### 3.3.8. Eukaryotic Initiation Factor 2 (eIF2) Pathway

eIF2 is one of the critical translation G proteins that are tightly regulated in the integrated stress response. In response to stress stimuli, eIF2*α* undergoes phosphorylation via different kinases, such as PKR-like endoplasmic reticulum kinase (PERK), which results in the formation of eIF2B. This blocks the p-eIF2 from its active GTP-bound state, causing a reduction of the translation of most mRNAs [71]. In the operative nervous systems, the eIF pathway was found to support the connectivity between neurons. The physiological extracellular cue Sema3A can trigger rapid and transient phosphorylation of eIF2a in axons for the axonal translational changes [72]. The D-MG group displayed the slightly elevated expression of eIF proteins, such as eIF-2*α*/EIF2S1 (0.220), eIF2*β*/EIF2S2 (0.274), and eIF2B proteins, such as EIF2B1 (0.43), EIF2B2 (0.47), EIF2B3 (0.45), and EIF2B5 (0.66) (Figure 8(d)), indicating potential stress such as hypoxia or insufficient nutrient uptake in the D-MG group. Also, the expression of EIF2AK3 (0.64), a stress sensing protein kinase that phosphorylates the alpha subunit of eIF2, was slightly elevated in the D-MG group. DDIT3 (0.58), which plays an essential role in inducing cell cycle arrest and apoptosis in response to ER stress, was also slightly elevated in the D-MG group. PPP1R15A (1.17) dephosphorylates the eIF-2*α*/EIF2S1, thereby reversing the shutoff of protein synthesis initiated by stress-inducible kinases and then facilitating cell recovery from stress.

#### 3.3.9. Extracellular Matrix

The extracellular matrix- (ECM-) related genes were found to be differentially expressed in the two groups (Supplementary [Supplementary-material supplementary-material-1]) [1]. The D-MG group had higher expression of some collagen genes including COL7A1, COL20A1, COL9A1, and COL11A2. The MG group expressed high levels of different types of collagens, including COL23A1, COL6A1, COL4A1, COL13A1, COL5A2, COL5A1, COL14A1, and COL4A1 [2]. The D-MG group mainly enriched laminin LAMB4, while the MG group enriched LAMA3, LAMB1, LAMC1, LAMA2, LAMA1, etc. [3]. The D-MG group expressed higher levels of specific integrins, including ITGA7, ITGB8, ITGA2, and ITGAB3, while the MG group enriched ITGA6, ITGA11, ITGA8, ITGB6, ITGA1, ITGB5, etc. [4]. For ECM remodeling genes, the D-MG group enriched proteases MMP17, MMP10, MMP24, MMP7, and MMP1; the MG group had higher expression of proteases MMP23, MMP2, MMP14, TIMP1, MMP28, MMP26, and MMP15, etc.

**Figure 9:**
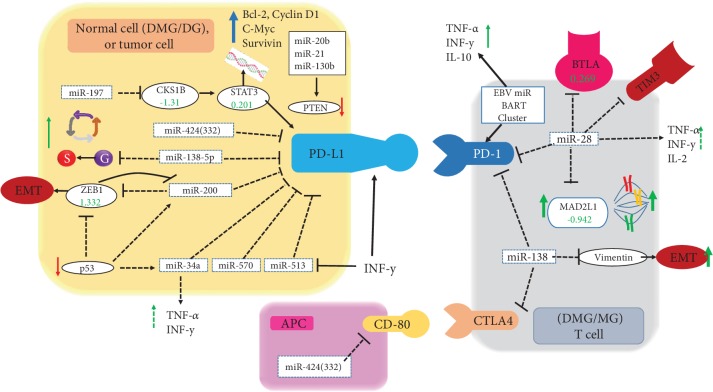
Illustration of microglia with PDL1/PD1 interactions. The numbers in the illustration are from the normal DMG/MG ratios. The arrows show the ways of tumor cell and T cell interactions. MGs act as antigen-presenting cells (APC).

Other pathways of our interests are shown in Supplementary [Supplementary-material supplementary-material-1], which include the AMPK pathway and PDL1/PD1 pathway (Figure 9 shows the relationship between miRNAs and PDL1/PD1 interactions) (Figure 9) [73]. Microglia, the main antigen-presenting cells in the human brain, maintain the equilibrium with T cells through the PD1 pathway as reported [74].

## 4. Conclusions

The genomics data reveal that the D-MG spheroids have higher expression of genes for glycolysis and hypoxia signaling, showing the metabolic shift to aerobic glycolysis, consistent with M1 polarization of microglia. However, glutamine conversion is not activated. The signaling pathway activities (activation of mTOR and p53, repression of NF-*κ*B and canonical Wnt) are consistent with the slower proliferation rate and the accumulation of differentiated cells. The additional NADPH needed for citrate and lipid synthesis is mainly generated by pentose phosphate pathway activation. The reduction in the proliferation rate allows the cells to achieve higher ATP levels in the spheroids. The D-MG group enriches genes for NOTCH signaling, but not canonical Wnt signaling. The MG group has higher expression of genes related to cell cycle and proliferation. These results can help to establish better coculturing methods for efficiently mimicking the *in vivo* structure of the central nervous system.

## Figures and Tables

**Table 1:** Glycolysis vs. oxidative phosphorylation.

Gene name	MG-1	MG-2	MG-3	D-MG-1	D-MG-2	D-MG-3	MG ave	DMG ave	DMG/MG	LOG2 (DMG/MG)
P4HA1	140.340	135.633	141.039	389.230	546.723	505.399	139.004	480.451	3.456	1.79
SLC2A1 (GLUT1)	579.1676	469.9516	562.8377	912.218	1098.52	1056.15	537.319	1022.293	1.903	0.93
DLST	17.881	18.114	17.362	26.470	26.133	24.754	17.786	25.786	1.450	0.54
SLC16A3	304.016	219.663	293.687	255.760	244.563	255.972	272.455	252.098	0.925	-0.11
COX4I1	238.629	185.922	239.199	186.813	225.728	199.922	221.250	204.154	0.923	-0.12
SLC25A1	72.139	59.151	72.118	54.289	60.972	56.058	67.803	57.107	0.842	-0.25
DLD	22.476	21.521	21.854	12.507	12.003	14.700	21.950	13.070	0.595	-0.75
OGDH	28.692	23.353	27.126	8.233	7.421	8.492	26.390	8.049	0.305	-1.71

The FPKM (fragments per kilobase per million reads) normalized values for these genes are listed for both samples. The numbers are the log2 values of ratios of D-MG to MG. Negative values indicate that the genes are present in higher amounts in the MG group, while positive values indicate that the genes are present in higher amounts in the D-MG group. The value 1 indicates two-fold change.

**Table 2:** Rate-limiting enzymes for central metabolic pathways and the ratios of their expression in the D-MG group compared to the MG group.

Pathway	Gene name	MG-1	MG-2	MG-3	D-MG-1	D-MG-2	D-MG-3	MG ave	DMG ave	LOG2 (DMG/MG)
Glucose phosphorylation	HK2	8.057	7.382	8.514	373.333	367.184	391.036	7.984	377.184	5.56
Glycolysis	PKM	144.193	130.476	142.160	1071.419	1269.244	1061.022	138.943	1133.895	3.03
Glycine synthesis	SHMT2	34.121	29.636	33.060	146.550	175.435	141.673	32.272	154.553	2.26
Asparagine synthesis	ASNS	28.779	28.424	28.878	93.603	122.579	101.086	28.694	105.756	1.88
Glycogenolysis	PYGB	25.047	22.544	24.888	34.525	34.766	30.397	24.159	33.229	0.46
Pyrimidine synthesis	CAD	8.199	9.554	8.489	13.220	12.638	10.185	8.747	12.015	0.46
Glycolysis	PFKL	62.436	52.127	63.963	83.298	79.339	73.566	59.509	78.734	0.40
Aspartate synthesis	GOT1	16.089	14.826	14.507	18.869	20.440	19.010	15.141	19.439	0.36
Fatty acid synthesis	FASN	28.814	22.644	29.725	36.264	34.796	31.673	27.061	34.244	0.34
Purine synthesis	PRPS1	23.085	20.259	22.740	24.847	28.698	27.976	22.028	27.173	0.30
Pentose phosphate	G6PD	15.355	12.245	15.422	12.237	14.129	12.097	14.341	12.821	-0.16
Fatty acid synthesis	ACAA1	21.219	19.891	21.847	17.973	18.352	15.681	20.986	17.336	-0.28
Glutamine-glutamate conversion	GLUD1	49.375	44.127	51.590	35.530	41.837	38.198	48.364	38.522	-0.33
Methionine synthesis	MTR	7.799	9.359	8.511	7.162	5.777	6.920	8.556	6.620	-0.37
TCA cycle	IDH2	103.753	84.981	101.212	66.326	79.889	75.170	96.649	73.795	-0.39
Serine synthesis	PHGDH	141.285	116.771	138.838	92.636	114.237	92.781	132.298	99.884	-0.41
Hexose	GFPT1	46.208	45.599	46.304	26.099	32.086	29.340	46.037	29.175	-0.66
Fatty acid oxidation	CRAT	23.955	20.886	25.945	14.810	14.697	14.392	23.596	14.633	-0.69
Proline synthesis	PYCR1	102.329	84.127	98.191	46.173	65.047	53.249	94.883	54.823	-0.79
Urea synthesis	CPS1	7.949	7.541	8.583	4.041	3.943	4.177	8.024	4.054	-0.99
Tyrosine synthesis	PAH	0.441	0.551	0.468	0.171	0.184	0.224	0.487	0.193	-1.33
Cysteine synthesis	MAT1A	5.443	4.897	5.280	0.793	0.724	0.529	5.207	0.682	-2.93

The FPKM (fragments per kilobase per million reads) normalized values for these genes are listed for both samples. The numbers are the log2 values of ratios of D-MG to MG. Negative values indicate that the genes are present in higher amounts in the MG group, while positive values indicate that the genes are present in higher amounts in the D-MG group. The value 1 indicates two-fold change.

**Table 3:** Sirtuin signaling and autophagy.

Gene name	MG-1	MG-2	MG-3	D-MG-1	D-MG-2	D-MG-3	MG ave	DMG ave	DMG/MG	LOG2 (D-MG/MG)
*Sirtuin signaling*										
SIRT6	11.268	9.088	9.289	20.250	21.520	17.741	9.882	19.837	2.007	1.01
SIRT1	8.625	8.714	9.529	12.048	12.060	13.258	8.956	12.455	1.391	0.48
SIRT4	3.945	5.113	3.877	5.457	4.254	5.735	4.312	5.148	1.194	0.26
SIRT7	7.837	8.696	7.100	8.127	7.686	7.362	7.877	7.725	0.981	-0.03
SIRT5	1.763	2.161	1.591	1.743	1.551	1.505	1.838	1.599	0.870	-0.20
SIRT3	13.160	13.397	12.523	8.864	7.755	8.300	13.027	8.306	0.638	-0.65
SIRT2	50.715	42.689	48.365	27.236	31.360	27.618	47.256	28.738	0.608	-0.72
*Autophagy*										
TFEB	5.357	4.069	5.025	4.723	5.425	5.416	4.817	5.188	1.077	0.11
BECN1	13.675	11.764	13.135	12.298	12.239	12.236	12.858	12.257	0.953	-0.07
LAMP1	57.074	48.954	58.426	46.114	51.499	47.309	54.818	48.307	0.881	-0.72

The FPKM (fragments per kilobase per million reads) normalized values for these genes are listed for both samples. The numbers are the log2 values of ratios of D-MG to MG. Negative values indicate that the genes are present in higher amounts in the MG group, while positive values indicate that the genes are present in higher amounts in the D-MG group. The value 1 indicates two-fold change.

**Table 4:** Gene expression of poly (ADP-ribose) polymerase (PARP) family proteins.

Gene name	MG-1	MG-2	MG-3	D-MG-1	D-MG-2	D-MG-3	MG ave	DMG ave	DMG/MG	LOG2 (D-MG/MG)
PARP6	19.908	24.159	20.683	55.689	59.154	54.018	21.583	56.287	2.608	1.38
PARP3	3.171	3.020	3.221	6.723	7.323	6.366	3.137	6.804	2.169	1.12
PARP2	17.659	22.634	16.142	35.570	39.811	34.828	18.812	36.736	1.953	0.97
PARP11	3.485	3.789	3.403	6.320	6.097	6.702	3.559	6.373	1.791	0.84
PARP15	0.163	0.124	0.127	0.286	0.263	0.179	0.138	0.243	1.759	0.81
PARP8	2.142	2.526	2.428	3.305	3.204	3.578	2.365	3.362	1.422	0.51
PARP1	42.914	40.611	42.447	41.423	43.048	40.686	41.990	41.719	0.994	-0.01
PARP10	1.451	1.234	1.709	1.518	1.198	1.385	1.465	1.367	0.933	-0.10
PARP16	12.871	12.281	11.812	7.840	8.730	9.558	12.321	8.709	0.707	-0.50
PARP4	8.746	9.413	8.348	3.874	3.104	3.526	8.836	3.501	0.396	-1.34
PARP14	7.616	10.770	8.298	1.482	1.613	1.818	8.895	1.638	0.184	-2.44
PARP12	1.519	1.168	1.288	0.182	0.274	0.228	1.325	0.228	0.172	-2.54
PARP9	3.633	3.688	3.397	0.533	0.460	0.586	3.573	0.526	0.147	-2.76

The FPKM (fragments per kilobase per million reads) normalized values for these genes are listed for both samples. The numbers are the log2 values of ratios of D-MG to MG. Negative values indicate that the genes are present in higher amounts in the MG group, while positive values indicate that the genes are present in higher amounts in the D-MG group. The value 1 indicates two-fold change.

## Data Availability

Data is available by contacting the corresponding author.

## Abbreviations

6PD: 6-Phosphate dehydrogenase
ACAA1: Acetyl-CoA acyltransferase
ACL: ATP citrate lyase
ACO2: Aconitase 2
ADP: Adenosine diphosphate
AIFM1: Apoptosis-inducing factor mitochondrion-associated 1
AKT: Protein kinase B
AMP: Adenosine monophosphate
AMPK: AMP-activated protein kinase
ANT: Adenine nucleotide translocase
APC: Antigen-presenting cells
ARF: ADP ribosylation factor
ASNS: Asparagine synthetase
ATP: Adenosine triphosphate
BAG2: Bcl-2-associated athanogene 2
BAX: Bcl-2-associated X
BCCIP*β*: BRCA2 and CDKN1A-interacting protein isoform *β*
Bcl-2: B-cell lymphoma 2
BCLAF1: Bcl-2-associated transcription factor 1
Bcl-XL: B-cell lymphoma-extra large
BECN1: Beclin 1
BMP4: Bone morphogenetic protein 4
BRN2: POU class 3 homeobox 2
CAD: Carbamoyl-phosphate synthetase 2
CAT: Catalase
CD11b: Integrin alpha M
CD45: Protein tyrosine phosphatase receptor type C
CDK: Cyclin-dependent kinase
CDK5RAP3: CDK5 regulatory subunit-associated protein 3
cDNA: Complementary DNA
CM: Cardiomyocytes
COL: Collagen
COX4-1: Cytochrome C oxidase 4 subunit 1
CPS1: Carbamoyl-phosphate synthase 1
CRAT: Carnitine O-acetyltransferase
CSL: Recombination signal binding protein for immunoglobulin kappa J region
CX3CR1: C-X3-C motif chemokine receptor 1
DAD1: Defender against apoptotic cell death
DDIT3: DNA damage inducible transcript 3
DLD: Dihydrolipoamide dehydrogenase
DLL: Delta-like canonical Notch ligand
DLST: Dihydrolipoyllysine-residue succinyltransferase
DMEM: Dulbecco's modified Eagle's medium
DMG/D-MG: Coculture of the dorsal forebrain spheroids with isogenic microglia-like cells
EC: Endothelial cells
ECM: Extracellular matrix
eIF2: Eukaryotic initiation factor 2
ENO: Enolase
Ep-iPSC: Episomal-induced pluripotent stem cells
ER: Endoplasmic reticulum
ERK: Extracellular signal-regulated kinases
FASN: Fatty acid synthase
FBS: Fetal bovine serum
FDR: False discovery rate
FGF: Fibroblast growth factor
FLT3L: FMS-like tyrosine kinase 3 ligand
FPKM: Fragments per kilobase per million reads
G6PD: Glucose-6-phosphate dehydrogenase
GALE: UDP-glucose 4-epimerase
GAPDH: Glyceraldehyde-3-phosphate dehydrogenase
GFPT1: Glucosamine-fructose-6-phosphate aminotransferase isomerizing 1
GLUD1: Glutamate dehydrogenase 1
GLUT1: Glucose transporter 1
GM-CSF: Granulocyte-macrophage colony-stimulating factor
GO: Gene Ontology
GOT: Glutamic-oxaloacetic transaminase
GPI: Phosphor-glucose isomerase
GSK-3*β*: Glycogen synthase kinase-3*β*
GTP: Guanosine triphosphate
HES: Hairy and enhancer of split 1
HIF-1*α*: Hypoxia-inducible-factor 1*α*
HINT: Histidine triad nucleotide-binding protein
hiPSCs: Human induced pluripotent stem cells
HK2: Hexokinase 2
IBA-1: Allograft inflammatory factor 1
IDH: Cytoplasmic isocitrate dehydrogenase
IDO: Indoleamine-pyrrole 2,3-dioxygenase
IkB: Nuclear factor of kappa light polypeptide gene enhancer in B-cell inhibitor
IL: Interleukin
ISR: Integrated stress response
ITG: Integrins
JAG: Jagged
KEGG: Kyoto Encyclopedia of Genes and Genomes
LAM: Laminin
LAMP1: Lysosomal-associated membrane protein 1
LDHA: Lactate dehydrogenase A
LDN: SMAD inhibitor LDN193189
LONP1: Lon protease homolog, mitochondrial
LPS: Lipopolysaccharide
LRP1: Low-density lipoprotein receptor-related protein 1
MAT1A: Methionine adenosyltransferase 1A
MAX: Myc-associated factor X
MDH1: Malate dehydrogenase 1
MDM2: Mouse double minute 2 protooncogene
MG: Microglia-like cells
MMP: Matrix metalloproteinase
MOMP: Mitochondrial outer membrane permeabilization
MPT: Mitochondrial permeability transition
MSC: Human mesenchymal stem cell
mTOR: Mechanistic target of rapamycin
MTR: 5-Methyltetrahydrofolate-homocysteine methyltransferase
Mxi-1: MAX-interacting protein 1
Myc: Myelocytomatosis
NADH: Nicotinamide adenine dinucleotide
NADPH: Nicotinamide adenine dinucleotide phosphate
NANS: Sialic acid synthase
NDUFA13: NADH dehydrogenase 1 alpha subcomplex subunit 13
NF-*κ*B: Nuclear factor kappa-B
NICD: Notch intracellular domain
NNT: NAD(P) transhydrogenase
OGDH: Oxoglutarate dehydrogenase
OXPHOS: Oxidative phosphorylation
P4HA1: Prolyl 4-hydroxylase subunit alpha 1
PAH: Phenylalanine hydroxylase
PAK4: p21-activated kinase 4
PARP: Poly (ADP-ribose) polymerase
PD1: Programmed cell death protein 1
PDH: Mitochondrial pyruvate dehydrogenase
PDK1: Pyruvate dehydrogenase kinase 1
PDL1: Programmed death-ligand 1
PERK: PKR-like endoplasmic reticulum kinase
PFK: Phosphofructokinase
PFKL: 6-Phosphofructokinase, liver type
PGAM5: Bcl-XL-binding protein v68
PGE2: Prostaglandin E2
PGK1: Phosphoglycerate kinase
PHD: Prolyl hydroxylase domain
PHGDH: Phosphoglycerate dehydrogenase
PIK3: Phosphoinositide 3-kinase
PKM: Pyruvate kinase isozymes M
PPID: Peptidyl-prolyl isomerase D
PPP1R15A: Protein phosphatase 1 regulatory subunit 15A
PPP2RA1: Protein phosphorylase 2A
PRDX3: Peroxiredoxin 3
PRMT1: Protein arginine methyltransferase 1
PRPS1: Phosphoribosyl pyrophosphate synthetase 1
PTBP1: Polypyrimidine tract-binding protein 1
PTP: Mitochondrial permeability transition pore
PTRH2: Bcl-2 inhibitor of transcription 1
PYCR1: Pyrroline-5-carboxylate reductase 1
PYGB: Glycogen phosphorylase B
qPCR: Quantitative polymerase chain reaction
RAC1: Ras-related C3 botulinum toxin substrate 1
Rheb: Ras homolog enriched in brain
ROS: Reactive oxygen species
RPS6KA: Ribosomal protein S6 kinase alpha
RT-PCR: Reverse transcription polymerase chain reaction
SATB2: Special AT-rich sequence-binding protein 2
SB: SMAD inhibitor SB431542
SCF: Stem cell factor
Sema3A: Semaphorin-3A
SHMT2: Serine hydroxymethyltransferase 2
SIAH2: Seven in absentia homolog 2
SIRT: Sirtuin
SLC16A3: Solute carrier family 16 member 3 (similar for SLC1A5, SLC25A1, and SLC38A2)
SREBP: Sterol regulatory element-binding protein
TBR1: T-box, brain 1
TCA: Tricarboxylic cycle
TCF: T cell factor/lymphoid enhancer factor
TFEB: Transcription factor EB
TGFBI: Transforming growth factor beta induced
TGF*β*R3: Transforming growth factor beta receptor 3
TIMP: Tissue inhibitor of metalloproteinases
UDP: Uridine diphosphate
UGDH: UDP-glucose 6-dehydrogenase
UGP2: UDP-glucose pyrophosphorylase
VDAC: Voltage-dependent anion channel
VEGF: Vascular endothelial growth factor
*α*-KD: *α*-Ketoglutarate dehydrogenase.

## Additional Points

*Significance*. Most current forebrain spheroids or organoids lack the components of microglia. This study uses the genomics tool to analyze various pathways of dorsal forebrain spheroids/organoids integrated with isogenic microglia-like cells derived from human induced pluripotent stem cells. The metabolic shift to aerobic glycolysis, consistent with M1 polarization of microglia, in the cocultured spheroids was observed. This study enhances our understanding of 3-D coculturing impacts on the metabolic programming of cells inside the forebrain spheroids. The results can be used to establish better coculturing methods for efficiently mimicking *in vivo* structure of the central nervous system.

## Supplementary Materials

Supplementary Materials Supplementary Table S1: primer sequence for target genes, Supplementary Table S2: genes related to ATP synthesis and mitochondrial complexes I, III, and IV. Supplementary Table S3: genes related to the NF-*κ*B pathway. Supplementary Table S4: AMPK pathway and PDL1/PD1 pathway. Supplementary Figure S1: microglial differentiation using additional human iPSC line: Ep-iPSC. Supplementary Figure S2: regulation of central metabolism in D-MG. Supplementary Figure S3: log2 gene expression levels of D-MG/MG ratios for ECM-related genes.
