# Supplementary material for: Genomics Analysis of Metabolic Pathways of Human Stem Cell-Derived Microglia-Like Cells and the Integrated Cortical Spheroids
Source: Stem Cells Int. 2019 Nov 18;2019:2382534. doi: 10.1155/2019/2382534 (PMC6885849; doi:10.1155/2019/2382534)

**Supplementary Materials**

**Genomics analysis of metabolic pathways of human stem cell-derived microglia-like cells and the integrated cortical spheroids**

Julie Bejoy^1, a^, Xuegang Yuan^1^, Liqing Song^1, b^, Thien Hua^2^, Richard Jeske^1^,

Sébastien Sart^3^, Qing-Xiang Amy Sang^2, 4^, Yan Li^1, 4,^ *

^1^Department of Chemical and Biomedical Engineering; FAMU-FSU College of Engineering; Florida State University; Tallahassee, FL USA

^2^Department of Chemistry and Biochemistry, Florida State University, Tallahassee, Florida, USA

^3^Hydrodynamics Laboratory (LadHyX) - Department of Mechanics; Ecole Polytechnique; CNRS-UMR7646; 91128 Palaiseau; France.

^4^Institute of Molecular Biophysics, Florida State University, Tallahassee, Florida, USA

^a^ Current address: College of Medicine, Vanderbilt University, Nashville, Tennessee, USA

^b^ Current address: Department of Chemical Engineering; Carnegie Mellon University, Pittsburgh, Pennsylvania, USA

**Supplementary Table S1. Primer sequence for target genes.**

| Gene | Forward primer 5' to 3' | Reverse primer 5' to 3' |
| --- | --- | --- |
| GLUT1  (SLC2A1) | AGCAACTGTGTGGTCCCTACG | AAGGTCCGGCCTTTAGTCTCA |
| PDK1 | AAACAGGGGAGCTTTGTCTGG | CTGCCCATTCACATCCCTCTA |
| HK2 | TGGTGTAGCTCCTCTGCTGCT | TGTGGGCACCCTTTAGTGAAC |
| HIF1A | GCATCTCCATCTCCTACCCACATAC | GGTGAGGCTGTCCGACTTTGAG |
| ERK1 | CCCTAGCCCAGACAGACATCTC | GGGCACAGTGTCCATTTTCTAAC |
| ERK2 | CCAGATTTGCTCTGCATGTGG | AGGTGAAGGTCTGAAGAACCACC |
| mTOR | GCCTGGATGGCAACTACAGAA | CCAGTTCAGCAAGGGGTCATA |
| NFK1B | GACGAGCTCCGAGACAGTGAC | GAGGCACCACTGGTCAGAGAC |
| PIK3CA | TTGGAGAACTTGGCCTTCATCT | ACCCAATTAGGTCTGAGGACTGAA |
| β-actin | GTACTCCGTGTGGATCGGCG | AAGCATTTGCGGTGGACGATGG |

**Supplementary Table S2. Genes related to ATP synthesis and mitochondria complex I, III, and IV.**

**Supplementary Table S3. Genes related to NF-kB pathway.**

**Supplementary Table S4. AMPK pathway and PDL1/PD1 pathway.**

**Supplementary Figure S1. Microglia differentiation using additional human iPSC line: Ep-iPSC.** Representative fluorescent images of CD45, CD11b, CD45/IBA-1, and P2RY12 (day 38). The co-cultured microglia cells and dorsal spheroids were indicated by β-tubulin III (green)/P2RY12 (red) expression. Blue: Hoechst 33342. White scale bar: 100 μm. White scale bar: 100 μm.

Human Ep-iPSC cells were obtained commercially from ThermoFisher (Cat #A18945). The Gibco Human Episomal iPSC Line was derived from CD34+ cord blood using a three-plasmid, seven-factor (SOKMNLT; SOX2, OCT4 (POU5F1), KLF4, MYC, NANOG, LIN28, and SV40L T antigen) EBNA-based episomal system. This iPSC line is considered to be zero foot-print as there was no integration into the genome from the reprogramming event and is free of all reprogramming genes. Human Ep-iPSC cells were maintained in StemFlex^TM^ Medium (ThermoFisher) on growth factor reduced Geltrex or Matrigel-coated surface. The cells were passaged by Versene (an EDTA-based solution, ThermoFisher) every 3-4 days and seeded at 1:8-1:12 ratio onto the new surface. Microglia differentiation was performed using the same method for iPSK3 line. Preliminary characterization was performed by immunocytochemistry for day 38 cells. Co-culturing of the derived microglia cells and dorsal spheroids were also performed at day 31, grown for another 7 days. The spheroids were replated and immunocytochemistry was performed.

**
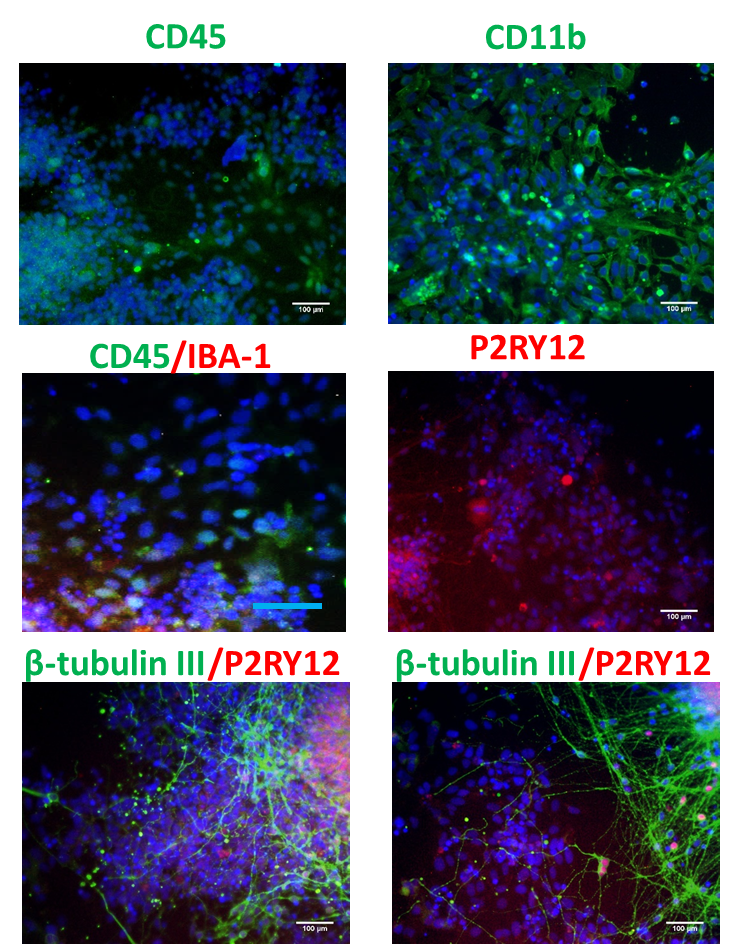
**

**Supplementary Figure S2. Regulation of central metabolism in D-MG.** Relative transcripts expression between D-MG and MG for (A) glycolysis, (B)TCA and ATP production, (C) glutamine and α-ketoglutarate metabolism, (D) NADPH and citrate metabolism. * indicates *p*<0.05 (n=3). (E) Schematic diagram showing the major metabolic changes in D-MG versus MG conditions: D-MG displays enhanced glycolysis, while TCA, glutamine, NADH and α-ketoglutarate metabolisms are down-regulated.

**
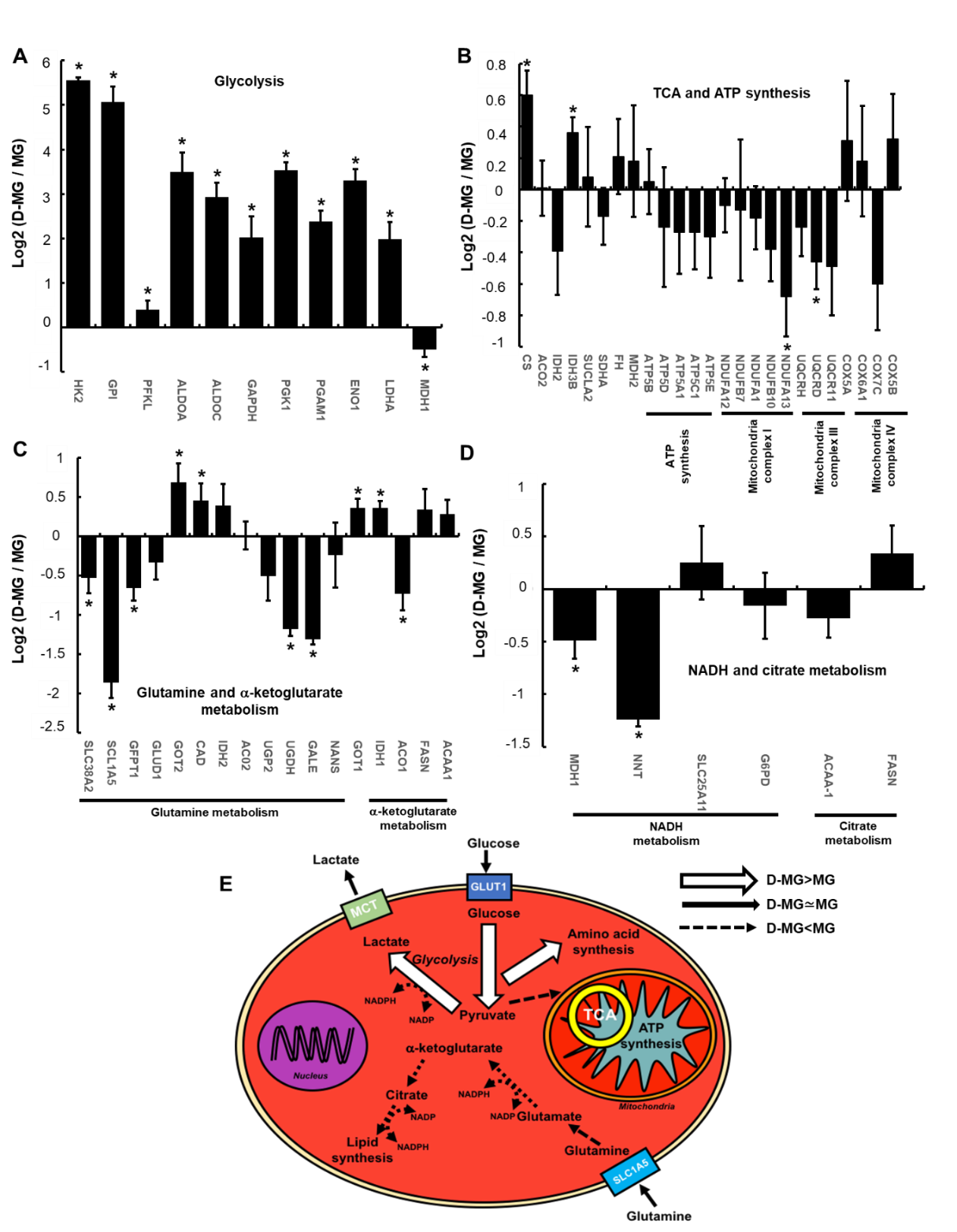
**

**Supplementary Figure S3. Log2 gene expression levels of D-MG/MG ratios for ECM-related genes.** (A) Collagens; (B) Laminins; (C) Integrins; (D) Extracellular matrix remodeling proteins.


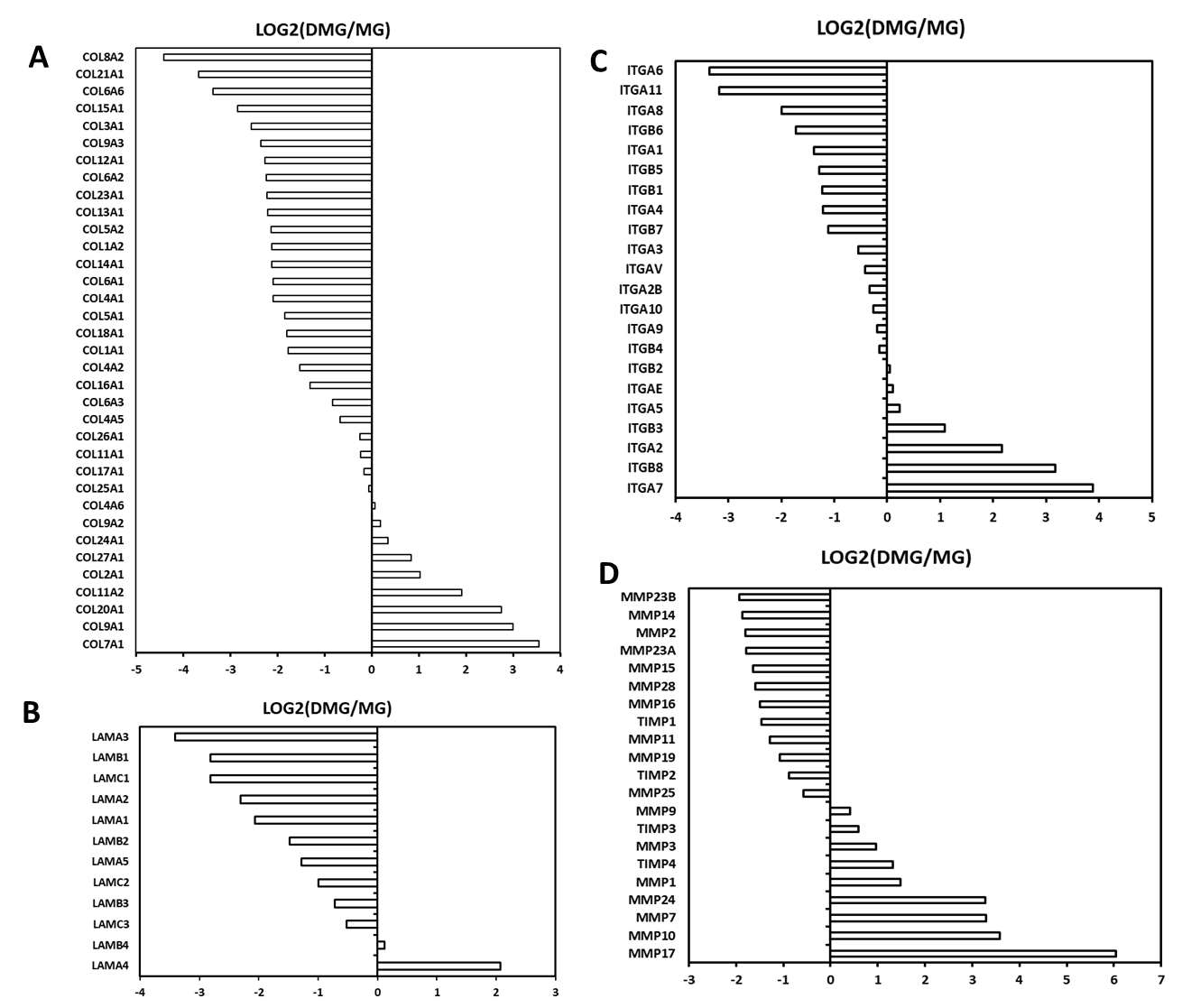

Supplement: Supplementary Materials — Supplementary Table S1: primer sequence for target genes, Supplementary Table S2: genes related to ATP synthesis and mitochondrial complexes I, III, and IV. Supplementary Table S3: genes related to the NF-κB pathway. Supplementary Table S4: AMPK pathway and PDL1/PD1 pathway. Supplementary Figure S1: microglial differentiation using additional human iPSC line: Ep-iPSC. Supplementary Figure S2: regulation of central metabolism in D-MG. Supplementary Figure S3: log2 gene expression levels of D-MG/MG ratios for ECM-related genes. [file 2382534.f1.docx]
